# Ion Chemistry of Carbon Dioxide in Nonthermal Reaction with Molecular Hydrogen

**DOI:** 10.1021/acs.jpca.2c01695

**Published:** 2022-05-31

**Authors:** Mauro Satta, Daniele Catone, Mattea Carmen Castrovilli, Paola Bolognesi, Lorenzo Avaldi, Nicola Zema, Antonella Cartoni

**Affiliations:** † Department of Chemistry, Institute of the Study of Nanostructured Materials-CNR (ISMN-CNR), Sapienza University of Rome, P. le Aldo Moro 5, Rome 00185, Italy; ‡ Institute of Structure of Matter-CNR (ISM-CNR), Area della Ricerca di Tor Vergata, Via del Fosso del Cavaliere, Rome 00133, Italy; § Institute of Structure of Matter-CNR (ISM-CNR), Area della Ricerca di Roma 1, Via Salaria km 29.300, Monterotondo 00015, Italy; ∥ Department of Chemistry, Sapienza University of Rome, P. le Aldo Moro 5, Rome 00185, Italy

## Abstract

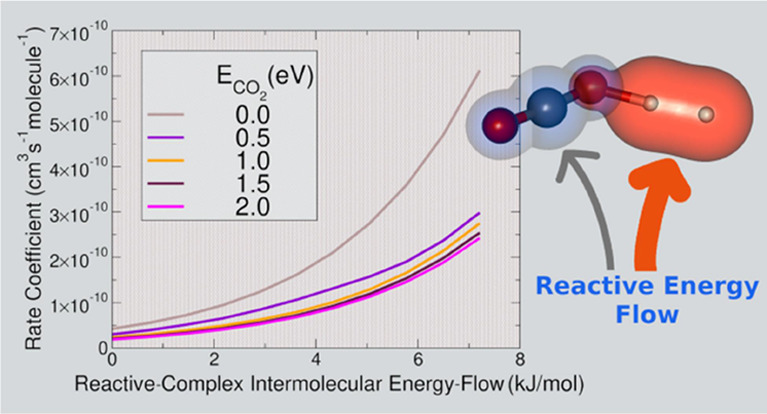

The exothermic hydrogen transfer from H_2_ to CO_2_^·^^+^ leading to H and HCO_2_^+^ is investigated in a combined experimental and theoretical work. The experimental mass/charge ratios of the ionic product (HCO_2_^+^) and the ionic reactant (CO_2_**^·^^+^**) are recorded as a function of the photoionization energy of the synchrotron radiation. Theoretical density functional calculations and variational transition state theory are employed and adapted to analyze the energetic and the kinetics of the reaction, which turns out to be barrierless and with nonthermal rate coefficients controlled by nonstatistical processes. This study aims to understand the mechanisms and energetics that drive the reactivity of the elementary reaction of CO_2_**^·^^+^** with H_2_ in different processes.

## 1 Introduction

Carbon dioxide, CO_2_, is one of the trace gases present in the Earth’s atmosphere and is uniformly distributed in its layers. This molecule is also the main component of the Mars and Venus atmospheres,^1,2^ and it is also present in the interstellar medium as neutral (CO_2_) and ionic (CO_2_^·^^+^, HCO_2_^+^) species.^3,4^ CO_2_ and other trace gases such as methane, CH_4_, nitrous oxide, N_2_O, and water, H_2_O, absorb infrared light coming from the Earth’s surface and consequently increase the temperature of the planet, producing the so-called “atmospheric greenhouse effect”. The increase of CO_2_ in the atmosphere is also the main factor for the increased acidity of the sea.^5^ The need for rapid reduction of fossil fuel emission and the requirement of “negative CO_2_ emissions” are pushing for the development of new approaches and technologies to produce energy (renewable sources)^6,7^ and remove CO_2_ from the atmosphere (carbon geoengineering).^8^ Indeed, CO_2_ conversion into suitable chemicals and fuels such as methanol, formaldehyde, and formic acid is one of the great challenges of the 21st century.^9^ The transformation of CO_2_ requires a coreactant that acts as a hydrogen source (like CH_4_, H_2_, or H_2_O) or the splitting of CO_2_ into CO and O_2_.^10^ Among the different emerging and novel technologies for the activation and conversion of CO_2_, the plasma-based approach has recently received much attention.^11^ In this strategy, carbon dioxide is activated by energetic electrons and the experimental conditions are mild, clean, and upscaling. Plasma is an ionized gas containing also neutral species, for example, atoms, molecules, radicals, and excited species that can emit light. All these species create a complex network of chemical reactions that can produce a system of interest for many potential different applications^12^ and, in the best cases, used on an industrial scale. The plasma is in “local thermodynamic equilibrium” (LTE) if all the species are at the same temperature (thermal plasma), which is otherwise called nonthermal plasma.^13^ Here, highly energetic electrons can activate the inert carbon dioxide^14^ at room temperature, which then can react with species such as CH_4_, H_2_O, or H_2_, leading to an efficient conversion of CO_2_ despite the low selectivity in the correspondent products. However, this undesired effect can be overcome by employing plasma catalysis, which can lead to a more selective production of specific compounds.^15^ Although there are several setups for the plasma-based CO_2_ conversion, the mechanistic insights into CO_2_ transformation are not well understood, and this prevents the prediction of a realistic trend on product yields and selectivity. Hence, an accurate experimental and theoretical analysis at the molecular level of the reactions of the “internally excited” CO_2_^·^^+^ with these neutrals is undoubtedly relevant.^16−21^ The reaction of carbon dioxide cation with molecular hydrogen has been already studied at low temperatures (15–300 K) under thermal conditions by Gerlich and co-workers.^22^ Instead, in the present study, we report an experimental and theoretical study of the same reaction with different internal energy of the carbon dioxide cation: the tunable synchrotron radiation has been used to excite vibrationally the reagent ions and the experimental results has been analyzed by developing a theoretical model based on the variational transition state theory (VTST).^23^ The VTST is a powerful tool to study barrierless reactions,^24^ and it can be modeled to study theoretical reactions under nonthermal conditions and with nonstatistical energy distribution among the different degrees of freedom. Specific theoretical models have been developed to describe nonstatistical reactions, and adaptations of statistical theories are of fundamental importance to explain specific experimental reactions.^25,26,17^

The results of this work unravel the mechanistic details of this ion-molecule reaction of multidisciplinary interest spanning from the nonthermal plasma technology for CO_2_ conversion to the chemical processes occurring in the space.

## 2 Methods

### 2.1 Synchrotron Experiments

The monochromatized radiation from the beamline CiPo (Circular Polarization) at ELETTRA (Trieste), described in detail in our previous studies,^27−30^ has been used to produce carbon dioxide radical cations with different internal energies. The beamline, equipped with an electromagnetic elliptical undulator/wiggler, works in the vacuum ultraviolet (VUV) region, and a normal incidence monochromator (NIM) provides photons in the 8–40 eV energy range. The aluminum grating of the NIM, operating in the energy range 8–17 eV and providing a photon flux of about 10^9^–10^10^ photon/s with an energy resolution of about 10–20 meV, has been used. The photon energy was calibrated against the autoionization features observed in the Ar total photoionization cross-section between the 3p spin orbit components.^31^ The effusive molecular beam of carbon dioxide has been introduced in the ion source through a leak valve and ionized by synchrotron radiation at a pressure of about 10^–6^–10^–5^ mbar. The generated CO_2_^·^^+^ ions were guided with several optical lenses into the octupole reaction cell at the nominal collision energy (CE) of 0 eV with an energy spread of about 150 meV. This value has been obtained by measuring the CO_2_^+^ yield as a function of the retarding field at the entrance of the octupole. The reactants H_2_ or D_2_ were inserted into the reaction cell (octupole) at different nominal pressures from 9.0 × 10^–6^ to 9.0 × 10^–5^ mbar and at room temperature. Mass spectra in the mass over charge (*m/z*) range 10–48 were acquired at 14.0 eV photon energy without and with neutral gases in the reaction cell at the nominal pressures of 9.0 × 10^–6^ and 3.0 × 10^–5^ mbar and with acquisition time ranging from 1 to 5 s/point. The areas of the signals acquired at *m/z* = 44 (CO_2_^·^^+^), 45 (HCO_2_^+^), and 46 (DCO_2_^+^) were fitted by Gaussian profiles with the OriginPro 2015 software to evaluate the isotopic effect. The intensities of the reagent and product ions in the ion-molecule reactions of CO_2_^·^^+^ with H_2_ or D_2_ were measured by scanning the photon energy in the range of 13.7–15.0 eV with a step of 0.10 eV and an acquisition time of 30 s/point at several pressure values in the reaction cell, namely, 0.9 × 10^–5^, 1.2 × 10^–5^, 3.0 × 10^–5^, 6.0 × 10^–5^, and 8.6 × 10^–5^ mbar. The pressure measurements are affected by an error of about 30%. For the sake of clarity, the ^·^ in CO_2_^·^^+^ is omitted in the following sections.

#### 2.1.1 Materials

All the samples were used at room temperature. Carbon dioxide CO_2_ was from SIAD with purity >99.99%. The H_2_ and D_2_ gases were purchased from Sigma-Aldrich with purity >99.99% and with a 99.8 atom % D.

### 2.2 Theoretical Calculations and Methodology

#### 2.2.1 Reactive Potential Energy Surface

The interaction between the ionized CO_2_^+^ and the neutral hydrogen molecule has been studied by means of ab initio calculations based on density functional theory (DFT) double-hybrid approach, which takes into account the radical nature of the species during the reaction. The functional used through all the calculations is the B2PLYP of Grimme,^32^ and the basis set is the valence double-zeta Pople polarization and diffuse functions 6-31++G**.^33^ All the frequency calculations have been carried on in the harmonic approximation. The electronic structure calculations have been done using the Gaussian code.^34^ The accuracy of the calculation has been verified by comparing the reaction enthalpy of 121.36 kJ/mol^35^ with the theoretical value of 121.9 kJ/mol obtained in this work. The open shell radical nature of the present reaction is properly accounted for with the employed level of calculation, as shown by the partial charge and spin analysis presented in the next section. The reactive potential energy surface (PES) has been calculated by scanning both the O–H and H–H coordinates with a variable step whose minimum value has been taken as 0.02 Å in the regions near the reactive complex. During the scan, all the other geometrical coordinates, except the scanning ones, have been optimized. The charge and spin population are based on the Mulliken analysis of the electron density.^36^ The geometries and the normal coordinates of the species are reported in the Supporting Information (SI) in Table S1–S3.

#### 2.2.2 Nonthermal Rate Coefficient

The VTST^23^ has been adopted to calculate the rate coefficient of the present reaction, following a nonthermal (NT) approach, which resembles the experimental conditions where the ionized CO_2_^+^ is not in thermal equilibrium with the H_2_ neutral molecule and the energy flow within the reactive complex does not follow a statistical distribution. Hence, the rate coefficient has been obtained by averaging over the translational and rotational energy, whereas the vibrational energy of CO_2_^+^ is not used in Boltzmann thermalization because its vibrational population is controlled by the amount of energy adsorbed during the photoionization process. The microcanonical nonthermal rate coefficient (*k*_μ_^NT^) is given by
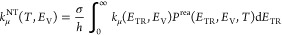
1where *k*_μ_ is the standard bimolecular microcanonical rate coefficient, *E*_V_ is the vibrational energy of CO_2_^+^, σ the rotational symmetry factor, and *E*_TR_ is the translational-rotational energy of the reactants (the translational energy is referred to the center of the mass frame in which the translational energy of the TS is zero). *P*^rea^ is the distribution probability of the reactants at temperature *T*:
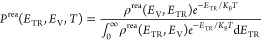
2and ρ^rea^ is the density of states of the reactants. We have considered only the vibrational energy of CO_2_^+^ because the vibrational partition function of H_2_ is unity for all temperatures of the present theoretical study (*T* ≤ 300 K) due to the high vibrational frequency of H_2_ (4381 cm^–1^).

The number of states of the reactants are
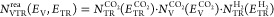
3where *N*_*V*_^H_2_^ is considered to be equal to 1 because at *T* ≤ 300 K, only the first vibrational level is populated due to the high vibrational frequency of H_2_.

Since ρ(*E*) = *∂N*(*E*)/*∂E*, the reactant density of state is:
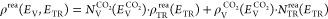
4where ρ^rea^_TR_(*E*_TR_) is the translational-rotational density of states for the reactants and is given by
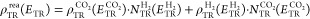
5and *N*_TR_^rea^ is the translational-rotational number of states of the reactants
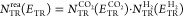


The integral at the denominator of eq 2 is
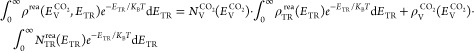
6

Equation 6 can be simplified by using the Laplace transform of the density and number of states:
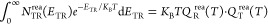

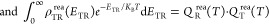

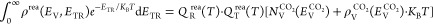
7where *Q*_R_^rea^(*T*) is the rotational and *Q*_T_^rea^(*T*) the translational molecular partition function of the reactants at temperature *T*.

The vibrational number of states is much greater than the density number of states up to *T* = 300 K; hence, the term ρ_V_^CO_2_^(*E*_V_^CO_2_^) · *K*_B_*T* in eq 7 can be neglected.

Equation 2 then can be rewritten as follows:
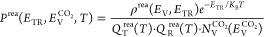
8where *E*_TR_ = *E*_TR_^CO_2_^ + *E*_TR_^H_2_^.

The microcanonical rate coefficient can be written as
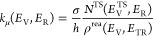
9where the number of states of the transition state (*N*^TS^), in the reference frame of the center of mass of the reactants, depends on the vibrational (*N*^TS^_V_) and rotational (*N*^TS^_R_) number of states:
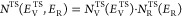
10

By substituting the eqs 8 and 9 into eq 1, the translational-rotational averaged nonthermal rate coefficient can be rewritten as:
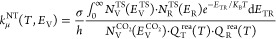
11and because , eq 11 became
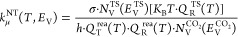
12

We consider TS as divided into two vibrational subsystems (a and b) not interacting with each other. System a is referred to the frequencies associated with the CO_2_^+^ modes not thermalized, and b to the intermolecular vibrations thermally averaged. Then, the vibrational number of states of TS can be written as
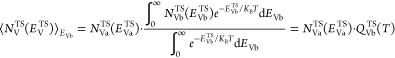
13

that gives the nonthermal rate coefficient:
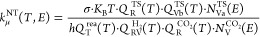
14where *Q*_R_^TS^(*T*) is the rotational partition function for the VTS complex, *Q*_T_^rea^(*T*) is the relative translation molecular partition function of the reagents, *Q*_RV_^H_2_^ is the rovibrational partition function of H_2_, *Q*_R_^CO_2_^ is the rotational partition function of CO_2_^+^, and *N*_V_^CO_2_^ is the number of vibrational states of CO_2_^+^. *Q*_Vb_^TS^ is the vibrational partition function of the VTS complex relative to the set *V*_b_, *N*^TS^_Va_ is the number of vibrational states of the VTS complex relative to the set *V*_a_. The number of vibrational states have been calculated by direct count (Beyer–Swinehart algorithm).^37^

Moreover, to calculate the rate coefficient of the reaction, it is relevant to know the energy, named *E*_TS_, acquired by the reactive complex when moving along the barrierless minimum energy path (MEP) from reagents to the VTS geometry. *E*_TS_ can be distributed among the internal degrees of freedom of the VTS complex as well as in the relative kinetic energy of the two products. The dynamics that control such energy “flow” are subtle and depend on several factors, such as the internal energy content of the reagents and the timescales of the internal vibration rearrangement (IVR) within the VTS complex. In thermal equilibrium conditions, the canonical rate coefficient is
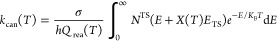
15where *Q*_rea_(*T*) is the molecular partition function for the reagents, *E* is the rovibrational energy of the system, *N*^TS^(*E*) is the rovibrational number of states of the VTS complex, and *X*(*T*) is the fraction of the *E*_TS_, which goes into all the degrees of freedom of the VTS complex, except that of the reaction coordinate. *X*(*T*) is defined as a parameter which controls the energy flow during the reaction. The increase in *X*(*T*) pushes the reaction energy flow toward the internal degrees of freedom of the reaction complex, vice versa, when *X*(*T*) goes to zero, all the reaction energy remains in the reaction coordinate and eventually goes into the relative kinetic energy of the products. Equation 15 can be solved to give the standard canonical form of the rate coefficient with an exponential part depending on the *X*(*T*)*E*_TS_:
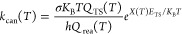
16

In the nonthermal condition of the present experiments, eq 16 cannot be applied, while eq 14 should be used after accounting properly the effect of *X*(*T*) within the VTS complex.

The nonthermal rate coefficient (eq 14) is transformed as follows:
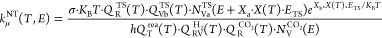
17where *X*_b_*·X*(*T*)·*E*_TS_ is the energy flow going into the intermolecular vibrations (*V*_b_) of the VTS complex, whereas the *X*_a_*·X*(*T*)·*E*_TS_ is the energy flow going into the vibrations (*V*_a_) of the VTS complex associated with CO_2_. The sum of *X*_a_ and *X*_b_ is always equal to 1.

## 3 Results and Discussion

**Figure 1:**
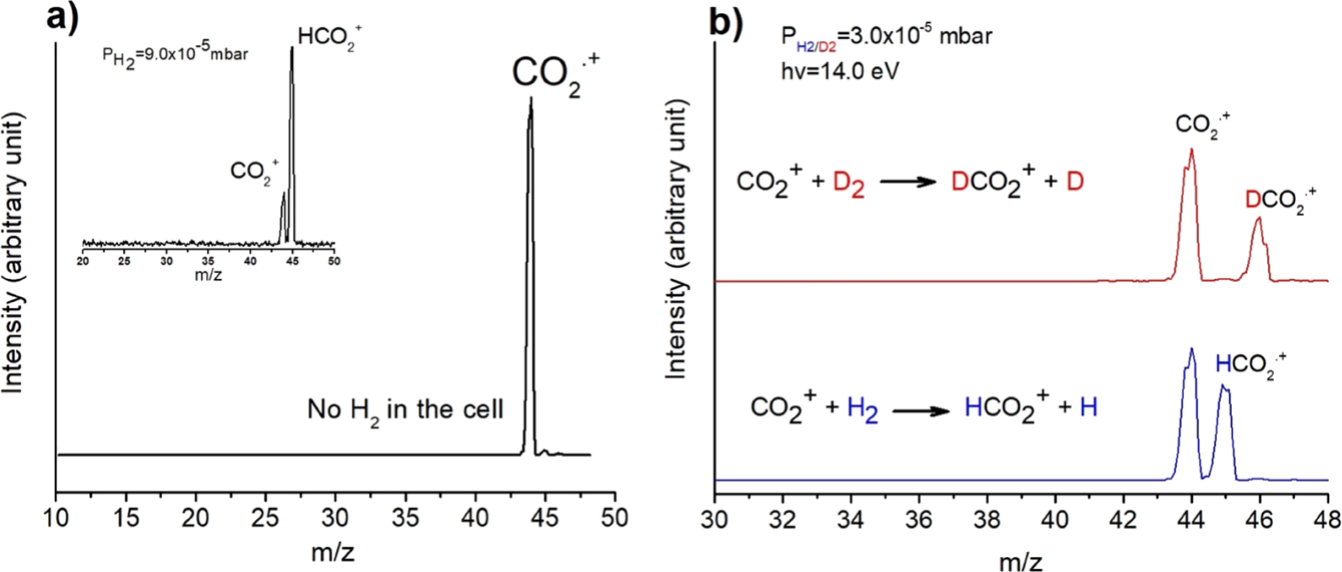
(a) Mass spectrum of CO_2_ at 14.0 eV photon energy. The CO_2_ pressure in the ion source was 1.8 × 10^–5^ mbar and no H_2_ gas in the reaction cell. In the inset, the mass spectrum acquired with H_2_ in the reaction cell at the nominal pressure of 9.0 × 10^–5^ mbar and nominal CE = 0 eV. (b) Comparison of the mass spectra acquired at the 14.0 eV photon energy, at a nominal pressure of about 3.0 × 10^–5^ mbar, and nominal CE = 0 eV for the reaction of CO_2_^+^ with D_2_ (red line) and H_2_ (blue line).

In the explored photon energy range from 13.7 to 15.0 eV, the carbon dioxide is ionized (ionization energy = 13.777 ± 0.001 eV)^38^ and vibrationally excited in its ionic ground state X^2^Π_3/2,1/2g_^39^ without dissociation, as several spectroscopic studies of CO_2_^+^ have demonstrated.^40^ The mass spectrum of CO_2_ measured at 14.0 eV photon energy in the range 10 < *m/z* < 48 is shown in Figure 1a, where neither fragment ions nor water traces are observed. The introduction of the H_2_ molecule in the reaction cell at a pressure of 9.0 × 10^–5^ mbar induces the hydrogen atom transfer (HAT) reaction 18 that generates the protonated form of carbon dioxide (inset in Figure 1a) HCO_2_^+^ detected at *m/z* = 45,
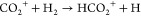
18

To evaluate the isotopic effect, the reaction was also performed with D_2_ and, as expected, a peak at *m/z* = 46 due to the CO_2_D^+^ ion was recorded. In Figure 1b, the spectra obtained at the photon energy 14.0 eV with H_2_ (D_2_) in the reaction cell at the nominal pressure of 3.0 × 10^–5^ mbar are shown. By fitting the area of the peaks at *m/z* = 44, 45 and 46, the obtained ratios CO_2_^+^/HCO_2_^+^ = 1.3 and CO_2_^+^/DCO_2_^+^ = 2.12 give an isotopic effect of 1.6.

**Figure 2:**
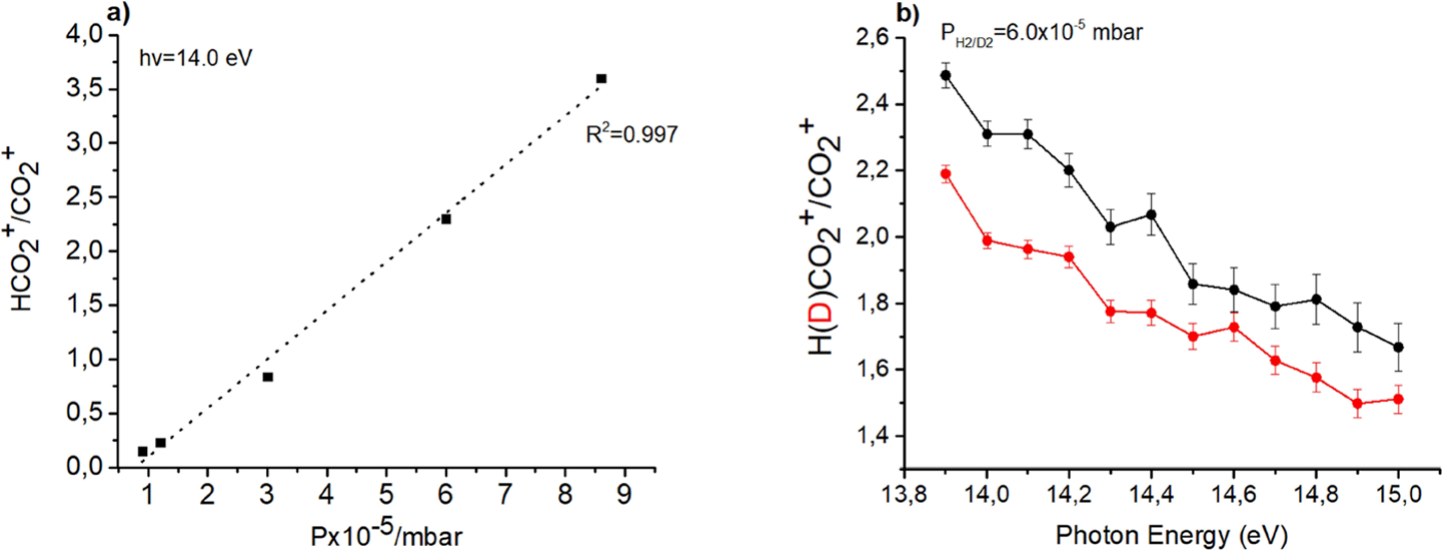
(a) Trend of HCO_2_^+^/CO_2_^+^ ratio vs H_2_ pressure in the CO_2_^+^/H_2_ ion-molecule reaction. (b) H(*D*)CO_2_^+^/CO_2_^+^ ratio vs photon energy for the reaction of CO_2_^+^ with H_2_ (black line) and D_2_ (red line) at nominal CE = 0 eV and at H_2_ (D_2_) nominal pressure of about 6.0 × 10^–5^ mbar in both cases.

The reactions were also studied at fixed photon energy (*hv* = 14 eV) to verify the linear increase of the H(*D*)CO_2_^+^/CO_2_^+^ ratio with the pressure of H_2_(D_2_) in the reaction cell (Figure 2a) and by scanning the photon energy from 13.7 to 15.0 eV to measure the H(*D*)CO_2_^+^/CO_2_^+^ ratio as a function of photon energy at a fixed H_2_(D_2_) pressure (Figure 2b).

The experimental data in Figure 2b show that the reaction is not favored by the increase of the vibrational energy of the CO_2_^+^. Literature data^41^ at room temperature (298 K) and at low pressure report rate coefficients *k* of 5.80 × 10^–10^ ± 10% and 4.10 × 10^–10^ ± 10% molecule^–1^ s^–1^ cm^3^ for the reaction with H_2_ and D_2_ respectively, whereas the rate coefficients reported by Gerlich and co-workers^22^ at high pressure are 9.5 × 10^–10^ ± 20% and 4.9 × 10^–10^ ± 20% molecule^–1^ s^–1^ cm^3^ for reaction with H_2_ and D_2_, respectively. Consequently the calculated isotopic effect (*k*_H_/*k*_D_) at low pressure is 1.4 ± 0.3 which is in quite good agreement with our low pressure experimental result of 1.6 obtained from data shown in Figure 1b. Moreover, the Langevin rate coefficients^42^*k*_L_ 1.53 × 10^–9^ and 1.09 × 10^–9^ molecule^–1^ s^–1^ cm^3^ for the reaction with H_2_ and D_2_, respectively, demonstrate that the reaction efficiency relative to collision rate, (*k*_H(D)_/*k*_L_), is about 38% at low pressure^41^ and it is 62% (*k*_H_/*k*_L_) and 45% (*k*_D_/*k*_L_) when Gerlich’s data are considered.^22^

The mechanism of this reaction is unveiled by the theoretical calculations performed at the DFT level of theory to compute the MEP, and by VTST to calculate the rate coefficients. All energies reported here after are corrected for zero point energy (ZPE).

**Figure 3:**
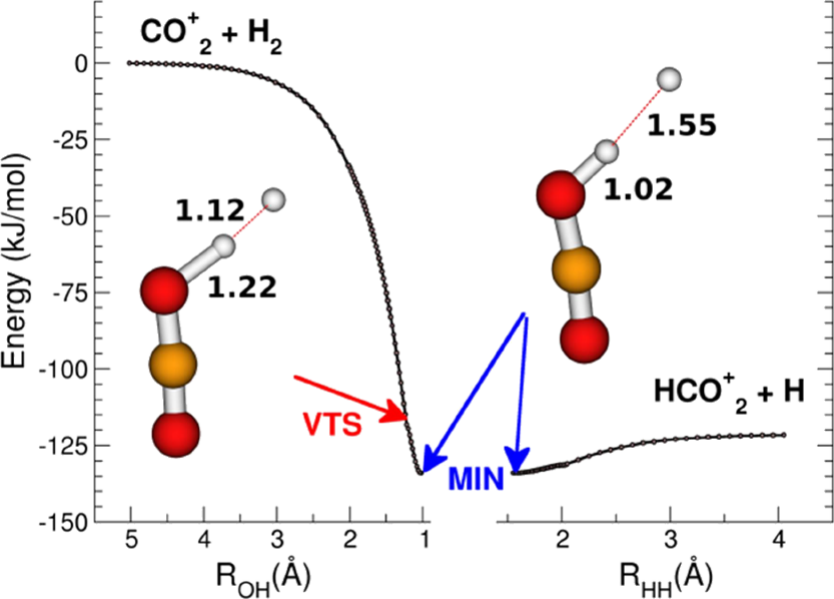
Minimum Energy Path of the reaction between CO_2_^+^ and H_2_ molecule. The zero energy is referred to the entrance channel of the reactants. The red arrow points to the geometry of the VTS Complex, whereas the blue arrow points to the minimum (MIN) of the MEP. The level of calculation is B2PLYP/6-31++G** with ZPE correction.

The MEP reported in Figure 3 shows that reaction 18 is barrierless, with a minimum (blue arrow) at −134 kJ/mol (−142 kJ/mol for D_2_) that has a dissociation energy of 12.1 kJ/mol (13.3 kJ/mol for D_2_) to form the products HCO_2_^+^ + H.

The reactive complex at MIN of the MEP has the two hydrogen atoms separated by 1.55 Å, while the interatomic distance between O and H is 1.02 Å. This structure shows that the reaction has almost already occurred with the atomic hydrogen weakly bound to the ionic product HCO_2_^+^, which remains quasi-linear, as well as the O–H–H group. From a dynamical perspective, the reaction proceeds with the H–H approaching one of the oxygen lone pair while the outgoing H atom bounces back along the direction over which the H–H has entered the reaction region. The geometry of the transition state is calculated by variational minimization of the number of vibrational states of the reactive complex along the reactive coordinate. In Figure 3, the position of VTS is indicated by a red arrow, and its energy is about 16 kJ/mol (19 kJ/mol for D_2_) higher than MIN, about 4 kJ/mol (5 kJ/mol for D_2_) above the energy of the products. In this VTS, the H–H bond is partially broken (1.12 Å), and the O–H bond is quasi-formed (1.22 Å).

**Figure 4:**
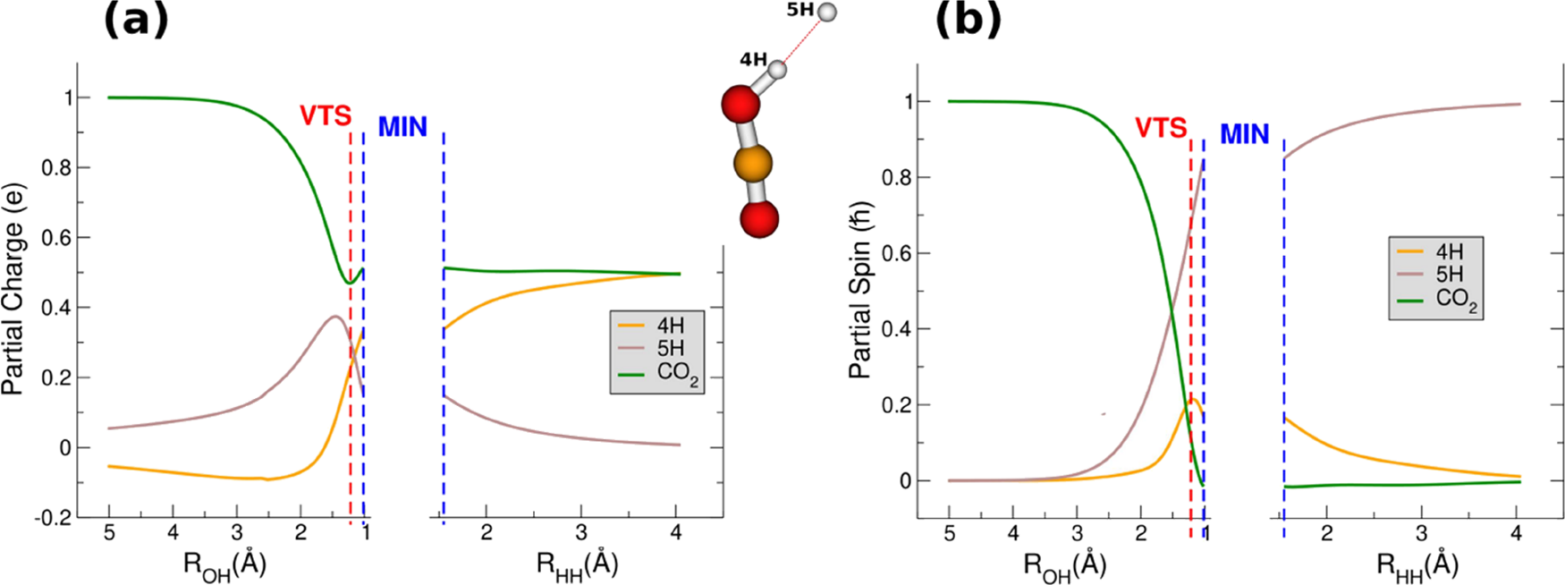
(a) Mulliken partial charges along the MEP. (b) Atomic partial spin along the MEP. Red dashed lines indicate the position of the VTS, while the blue dashed lines point to the MIN complex. 4H is the H bound to the oxygen atom and 5H the outgoing hydrogen atom.

The electronic nature of the VTS can be analyzed in terms of the partial charges *q* (see Figure 4a), where the outgoing hydrogen atom (5H) has a *q* of 0.3*e*, while the *q* value of CO_2_ is 0.47*e*, which is almost the value (0.5*e*) it reaches in the final products.

It is noteworthy that the positions along the MEP of the minimum of the *q* of CO_2_, as well as of the maximum of the *q* of the outgoing H, are very close in the VTS: the partially positive charged outgoing hydrogen (5H) has to be filled with half an electron charge to became neutral and to reach the final product region. The partial spins of the reactive systems are also interesting especially when considering the hydrogen atom which is transferred from H_2_ to CO_2_^+^: in the reactant region as well as in the final product region, this hydrogen has a zero net value of its partial spin; meanwhile, a maximum value of about 0.2*ℏ* is reached in the neighborhood of the VTS.

From a dynamical point of view, the maximum of the partial charge of the outgoing hydrogen and the corresponding maximum of the partial spin of the “transferred” hydrogen are correlated to a slowing down of the speed of the reaction: in this region of the MEP, where the VTS is located, the system decelerates its way toward the products. The break of the strongly bound H_2_ molecule is the key factor in the determination of the rate coefficient and the electron density of both H atoms is strongly shaken up during the reaction.

As for the energetics of the reaction, the low-pressure experimental conditions of the present study are such that the CO_2_^+^ and the VTS complex are not in thermal equilibrium in their internal degrees of freedom as well as with the surrounding molecules. The hydrogen molecule is at room temperature, as well as the roto-translation degrees of freedom of the CO_2_^+^ ion. Instead, the CO_2_^+^ vibrational states are excited by the energy absorbed during the photoionization and hence, not in thermal equilibrium with the surrounding. Furthermore, the H_2_ molecule plays a peculiar role with its high vibrational energy (4381 cm^–1^), which is poorly coupled with the other lower energy vibrations of the VTS complex, leading to a nonthermalized system. Hence, the vibrations of the transition state (see Table S4 in the Supporting Information) have been classified according to the following scheme: four frequencies form the set (*V*_a_) associated with the CO_2_ ion (578, 608, 1283, 2386 and578, 585, 1270, 2383 cm^–1^ in the reaction with H_2_ and D_2_, respectively), the negative frequency at −245 cm^–1^ for H_2_ (−181 cm^–1^ for D_2_) is associated with the reactive coordinate, and the other four vibrations (311, 909, 1068, 1170 and 232, 650, 776, 882 cm^–1^ in the reaction with H_2_ and D_2_, respectively) are classified as the set (*V*_b_) of intermolecular modes. These last frequencies are not considered when computing the number (*N*) of vibrational states of the VTS complex, which depends only on the frequencies associated with the CO_2_^+^ ion excited in photoionization, whereas the intermolecular vibrational modes of the VTS are considered to compute the corresponding vibrational molecular partition function (see eqs 13 and 14).

**Figure 5:**
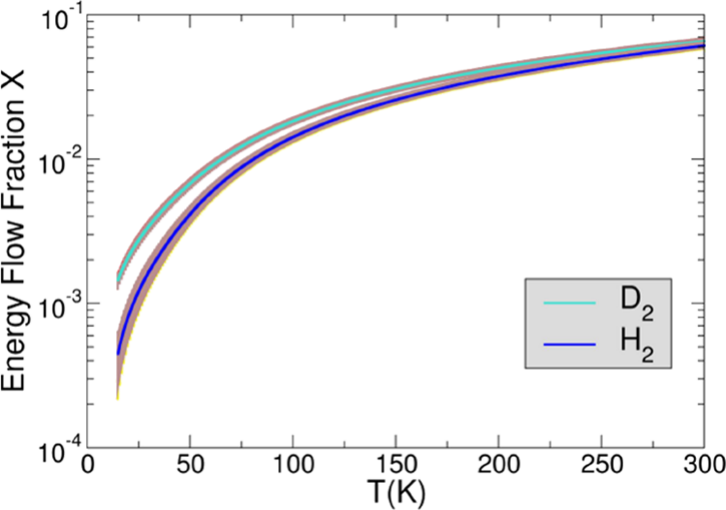
Energy flow fraction *X*(*T*) versus temperature. A fraction *X* of the energy *E*_TS_(*XE*_TS_) is transferred to the vibrational degrees of freedom of the TS. In blue is reported the energy fraction obtained by using the experimental rate coefficients for H_2_ measured by Gerlich and co-workers.^22^ The brown area shows the uncertainty due to the 20% error on the experimental rate coefficients. The cyan line represents similar data for reaction with D_2_.

*E*_TS_ is the difference between the energy of VTS (*E*_VTS_) and reagents (*E*_rea_) and its values are −118.2 and −124.0 kJ/mol for H_2_ (Figure 3) and D_2_, respectively. In order to obtain the fraction *X* of *E*_TS_ (see eq 16), which goes into the VTS complex, we used the experimental reaction rates obtained by Gerlich and co-workers in the temperature range between 15 and 300 K for reaction 18.^22^ By equating eq 16 with the *k*(*T*) obtained by Gerlich and co-workers,^22^ the fraction *X*(*T*) as a function of *T* can be derived, and it is shown in Figure 5.

*X*(*T*) at 15 K is 4.5 × 10^–4^ and 14.5 × 10^–4^ for H_2_ and D_2_, respectively, showing that at low temperature, the E_TS_ remains along the reaction coordinate and goes to the relative kinetic energy of the products. *X*(*T*) slowly increases with temperature, up to 6.1 × 10^–2^ and 6.6 × 10^–2^ for H_2_ and D_2_, respectively, at room temperature. The *X*(*T*) obtained in the temperature range 15–300 K has been fitted with the function *X*(*T*) whose parameters are reported in Table 1.

**Table 1:** Parameters of the Function *X*(*T*) = α*e*^–β*T*^–γ^^ + δ for the Reaction of CO_2_^+^ with H_2_ and D_2_. (*T* is in K)

	α	β	γ	δ
H_2_	1.7667	22.4123	0.3320	3.3205 × 10^–4^
D_2_	1.5483	19.4790	0.3176	1.2225 × 10^–3^

**Figure 6:**
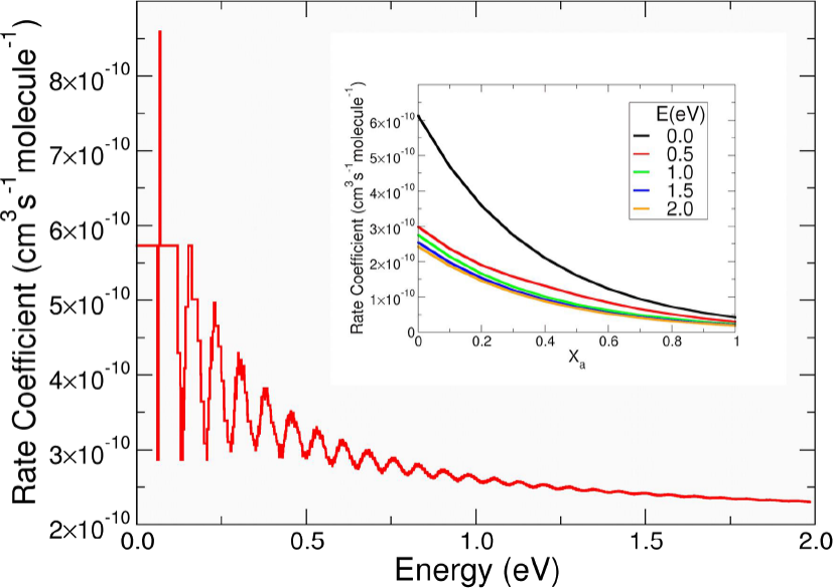
Rate coefficients as a function of the internal energy of CO_2_^+^ when vibrational energy distributions inside the VTS has a “*X*_a_*X*_b_ pair” equal to 0.1 and 0.9. The data are referred to *X*(300 K) = 0.0612. In the inset are reported the rate coefficients for different values of the internal energy of CO_2_^+^ and as a function of *X*_a_. See further details in the main text.

Hence, we have calculated the nonthermal rate coefficients (eq 17) at different energies for several values of the “*X*_a_*X*_b_ pair” at room temperature (300 K) when *X*(300 K) = 0.0612. Figure 6 reports such calculations for H_2_ (Similar data for the reaction with D_2_ are reported in Figure S2 of the Supporting Information) when *X*_a_ is 0.1 and *X*_b_ 0.9.

**Figure 7:**
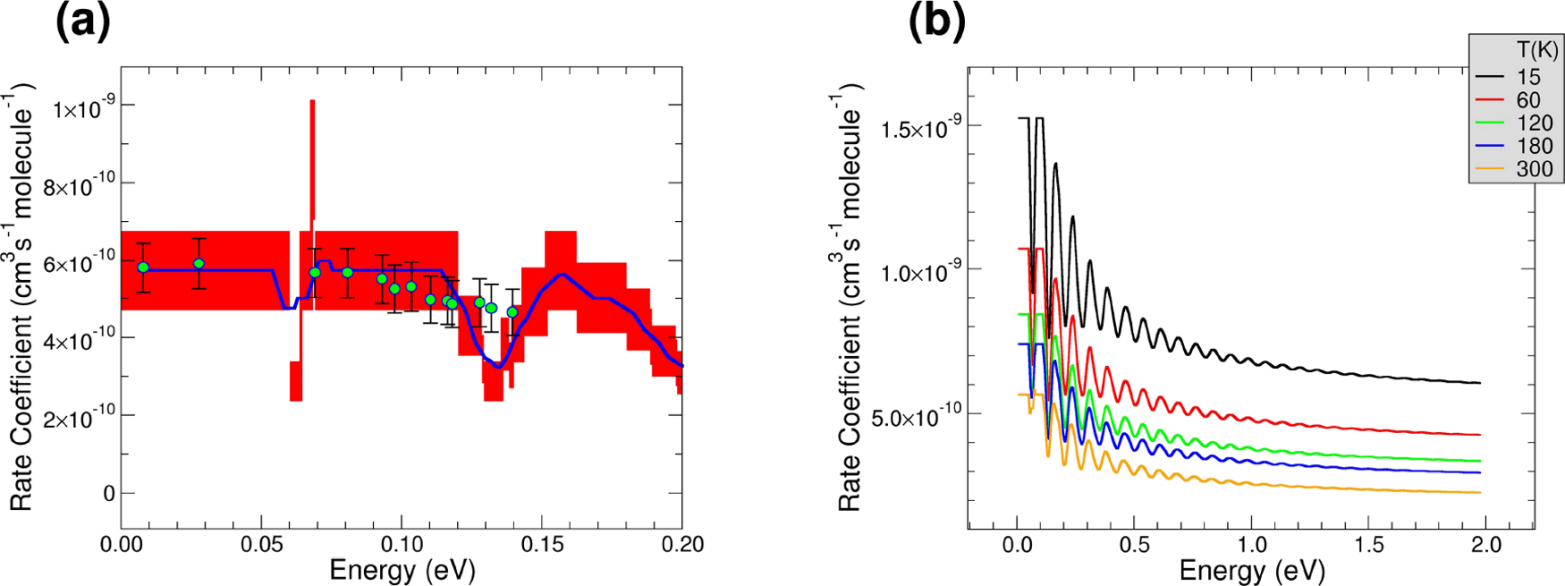
(a) Theoretical and experimental rate coefficients as a function of the internal energy of the system. The green points represent the experimental data, while the blue line is the theoretical rate coefficients calculated when *X*(300 K) = 0.0612 and *X*_a_ = 0.1. The red area shows the uncertainty due to the 20% error on the experimental rate coefficient from Gerlich.^22^ See main text for further details; (b) rate coefficients for the hydrogen transfer reaction between CO_2_^+^ and H_2_ as a function of the internal energy acquired during the photoionization of the CO_2_^+^ at different temperature *T*. *T* is the temperature of the roto-translations of the CO_2_^+^ and of the vibro-roto-translation of the H_2_ reagent. *X*(300 K) = 0.0612, and *X*_a_ = 0.1.

Rate coefficients presented in Figure 6 show that the reaction slows down when the internal energy increases and that below 1.0 eV the rate coefficients oscillate because of a competition between the population of the vibrational levels of CO_2_^+^ and that of the VTS complex. The increase in the population of the vibrations of the VTS complex produces an increase in the rate coefficient, while the increase in the population of the vibrations of the CO_2_^+^ induces a decrease in the rate coefficients. The rate coefficient is higher when the energy flow goes to the intermolecular vibrations (*V*_b_) of the VTS complex without exciting the vibrations of the VTS complex associated with CO_2_^+^ normal modes (*V*_a_) (see the low values of *X*_a_ in the inset of Figure 6). The rate coefficient decreases up to below 10^–10^ cm^3^ s^–1^ molecule^–1^ when the energy flow does not go to the intermolecular vibrations (*V*_b_) of the VTS complex (see the high values of *X*_a_ the inset of Figure 6). This trend can be rationalized considering how the vibrations that mainly favor the reaction are those associated with the formation of the O–H bond, and hence, all the four intermolecular vibrations (*V*_b_) of the VTS complex. Vice versa when the energy flow goes to the four vibrations (*V*_a_) of the VTS complex that come from CO_2_^+^, then the reaction is not favored because these vibrations are not relevant for the hydrogen transfer from H_2_ to CO_2_^+^. From a theoretical point of view, it is not so simple to provide a quantitative scenario describing the real energy flow between the two vibrational subsystem *V*_a_ and *V*_b_ of the VTS complex even if it could be expected that low values of *X*_a_, and complementary high values of *X*_b_ should correspond to the real dynamical description of the reaction. In order to verify such hypothesis, we have transformed the HCO_2_^+^/CO_2_^+^ ratio vs photon-energy data of Figure 2b in rate coefficients vs internal energy, following the procedure described in the paragraph 3 of the Supporting Information. The average internal energy of the CO_2_^+^ ion as a function of photon energy (Figure S1) allows to plot the experimental rate coefficients as a function of the internal energy of the CO_2_^+^ ion, and hence to compare the theoretical and experimental rate coefficients (see Figure 7a for H_2_ and Figure S3 for D_2_).

It is noteworthy that the “*X*_a_*X*_b_ pair” is a relevant parameter to select a theoretical rate coefficient in agreement with the experimental data. The *X*_a_(*X*_b_) = 0.1(0.9) is the best pair of parameters which is able to correctly reproduce the reaction kinetic at *T* = 300 K for reaction with H_2_, whereas the *X*_a_(*X*_b_) = 0.0(1.0) seems to better reproduce the reaction with D_2_ (see Figure S3 of the Supporting Information). In terms of energy flow, this means that for the reaction with H_2_ (*E*_TS_ = −118.2 kJ/mol), only *X*_a_*·X*(300 K)·*E*_TS_ = 0.7 kJ/mol is the energy that flows into the “CO_2_-like” vibrational levels (*V*_a_) of the VTS complex, while *X*_b_*·X*(300 K)·*E*_TS_ = 6.5 kJ/mol is the energy that goes to the intermolecular vibrations (*V*_b_) of the VTS complex. For the reaction with D_2_ (*E*_TS_ = −124.0 kJ/mol), all the energy goes to the intermolecular modes of the VTS, and *X*_b_*·X*(300 K)·*E*_TS_ = 8.1 kJ/mol. Hence, the relative kinetic energy of the two products H and HCO_2_^+^ is 111.0 kJ/mol, while for D and DCO_2_^+^ it is 115.9 kJ/mol. This means that the reaction occurs so quickly that there is no much time to share the reaction energy with the internal degrees of freedom of the VTS complex.

Because of the small amount of energy *X*(*T*)·*E*_TS_ transferred to the VTS complex at *T* = 300 K, which will be even smaller at lower *T* (see Figure 5), it is reasonable to assume that the “*X*_a_*X*_b_ pair” is independent from the temperature itself. Hence, in the temperature range between 15 and 300 K, where the *X*(*T*) is known, the rate coefficients can be calculated (see Figure 7b for H_2_ and Figure S4 for D_2_). The rate coefficients increase when the *T* decreases (see Figure S5 of the Supporting Information), and at *T* = 15 K, the reaction accelerates up to about 1.5 × 10^–9^ molecule^–1^ s^–1^ cm^3^ for H_2_ reaction (the collision limit), keeping the decreasing oscillating trend with energy at every *T* between 15 and 300 K (see Figure 7b). The general decreasing trend of the rate coefficient with the increase in the vibrational energy of CO_2_^+^ has the same decreasing behaviors of the Gerlich’s rates with temperature, where only the populations of the translational and rotational levels are involved. Hence, this reaction is not favored when either vibrational or roto-translational energy content are increased as generally expected in the barrierless reactions.

For reaction with D_2_, the temperature trend of the rate coefficient (see Figure S6 of the Supporting Information) has a maximum at about 55 K for all the energies up to 2 eV, and *k* = 6.5 × 10^–10^ molecule^–1^ s^–1^ cm^3^ is reached for *E* = 0 eV. The reason for the different temperature trend of the rate coefficients for H_2_ and D_2_ is the different fraction *X*(*T*): for both reactions, *X*(*T*) decreases with the temperature, but in the reaction with D_2_, the energy flow fraction at low *T* is greater than the one for the reaction with H_2_ (see Figure 5). Hence, the exponential part of eq 17 (), which makes the rate coefficient decreasing with decreasing *T*, is more effective in decreasing the rate coefficient below 55 K for the reaction with D_2_ then in the case of the reaction with H_2_. The rate coefficients for D_2_ at low temperatures have a different behavior in the present work with respect to thermal Gerlich’s data, and this can be explained with the nonstatistical energy distribution in the experimental conditions in our work.

## 4 Conclusions

The reaction of CO_2_^+^ with hydrogen molecules has been studied as a function of the CO_2_^+^ internal energy by using tunable synchrotron radiation to perform photoionization mass-resolved experiments, which have been analyzed and rationalized by means of the energetic and kinetics theoretical model. The reaction proceeds via hydrogen transfer from H_2_ to CO_2_^+^ with HCO_2_^+^ and H as final products. The experimental ratio of the charged product over the charged reactant shows a decrease in the reaction rate with increasing photon energy, confirmed also by the reaction of CO_2_^+^ with D_2_. The DFT minimum energy path of the reaction is barrierless and exothermic, and the variational transition state is located near the minimum of the energy along the reaction coordinate. Charge and spin population analysis clearly marks a strong reshuffle of the electron density when the reactive complex reaches the VTS geometry. The rate coefficient has been evaluated taking into account the nonthermal experimental conditions because of the low pressures in the reaction chamber. Moreover, the kinetic calculations considered the decoupling of the high energy vibration of H_2_ with respect to the lower energy vibrations of CO_2_^+^, and this is reflected in two vibrational sets of the VTS complex, which give different contribution to the overall reaction rate. The energy produced along the reaction coordinate is parameterized in terms of flows toward either the relative kinetic energy of the products or the internal degrees of freedom of the reactive complex. The present theoretical model gives an interpretation of the experimental data in terms of energy flow, revealing a decreasing trend of the rate coefficients with the photoionization energy. Furthermore, calculations of the reaction rates at different temperatures of the reactants confirm that the reaction at lower temperature reaches almost its Langevin upper limit of 1.53 × 10^–9^ molecule^–1^ s^–1^ cm^3^ . This study provides kinetic information that deserves to be considered within the network of the chemical reactions occurring where CO_2_ and H_2_ are present.

## Supporting Information Available

Data reported: normal coordinates at the B2PLYP/6-31++G** level of theory for reactive complex in its VTS geometry in the reaction of CO_2_^+^ with both H_2_ and D_2_; cartesian coordinates of reagents, products and VTS; normal mode analysis of the vibrational frequencies of the VTS geometry; average internal energy distribution upon photoionization; rate coefficients for the reaction of CO_2_^+^ with H_2_ and D_2_ in a range of *X*_a_*X*_b_ pair; rate coefficients versus temperature of the reactants (PDF)
