# Supplementary material for: Ion Chemistry of Carbon Dioxide in Nonthermal Reaction with Molecular Hydrogen
Source: J Phys Chem A. 2022 May 31;126(22):3463–71. doi: 10.1021/acs.jpca.2c01695 (PMC9189832; doi:10.1021/acs.jpca.2c01695)
Supplement: Supplementary file 1 — jp2c01695_si_001.pdf [file jp2c01695_si_001.pdf]

Supporting Information

The Ion Chemistry of Carbon Dioxide in NonThermal Reaction with Molecular Hydrogen

Mauro Satta,[1] Daniele Catone,[2] Mattea Carmen Castrovilli,[3] Paola Bolognesi,[3] Lorenzo Avaldi,[3] Nicola Zema,[2] Antonella Cartoni[4 *]*

1) Istituto per lo Studio dei Materiali Nanostrutturati (ISMN-CNR) Dipartimento di Chimica, Sapienza Università di Roma P.le Aldo Moro 5, 00185, Roma (Italy)

2) Istituto di Struttura della Materia (ISM-CNR), Area della Ricerca di Roma 2, via del Fosso del Cavaliere 10, 00133, Roma, Italy

3) Istituto di Struttura della Materia (ISM-CNR), Area della Ricerca di Roma 1, via Salaria Km 29,300, Monterotondo Scalo (RM), 00016, Italy.

4) Dipartimento di Chimica, Sapienza Università di Roma, Pl.e Aldo Moro 5, 00185, Roma, Italy.

Corresponding Authors

*A. Cartoni: E-mail: antonella.cartoni@uniroma1.it; Tel. + 39 06 49913678

*M. Satta: E-mail: mauro.satta@cnr.it; Tel. +39 06 49913381

Table of Contents:

1. Normal Coordinates and Geometries at the B2PLYP /6-31++G** Level of Theory (Table S1 to S3) S3
2. Normal Mode Analysis of the Vibrational Frequencies of the VTS Geometry (Table S4) S5
3. Average Internal Energy Distribution upon Photoionization S6
4. Rate Coefficients for the Reaction of CO_2^+ with H_2 and D_2 in a range of X_aX_b pair S7
5. Rate Coefficients versus Temperature of the Reactants S9

1. Normal Coordinates and Geometries at the B2PLYP /6-31++G** Level of Theory (Table S1 to S3):

Table S1. *Normal coordinates for reactive complex in its VTS geometry (reaction with H_2)*

	X	Y	Z	X	Y	Z	X	Y	Z
	-246 cm^{-1}			311 cm^{-1}			578 cm^{-1}		
1C	-0.02	0.01	0.00	-0.05	0.00	0.00	0.00	0.00	0.86
2O	0.03	0.00	0.00	0.06	0.05	0.00	0.00	0.00	-0.34
3O	-0.05	-0.02	0.00	-0.07	0.00	0.00	0.00	0.00	-0.29
4H	0.56	0.68	0.00	-0.13	-0.36	0.00	0.00	0.00	-0.02
5H	0.04	-0.47	0.00	0.83	-0.39	0.00	0.00	0.00	-0.23
	608 cm^{-1}			909 cm^{-1}			1068 cm^{-1}		
1C	0.58	0.24	0.00	0.00	0.00	0.02	0.00	0.03	0.00
2O	-0.22	-0.06	0.00	0.00	0.00	0.00	-0.02	0.06	0.00
3O	-0.20	-0.10	0.00	0.00	0.00	0.02	0.04	-0.01	0.00
4H	-0.50	0.11	0.00	0.00	0.00	-0.88	0.19	-0.54	0.00
5H	0.27	-0.42	0.00	0.00	0.00	0.47	-0.49	-0.65	0.00
	1170 cm^{-1}			1283 cm^{-1}			2386 cm^{-1}		
1C	-0.05	0.00	0.00	0.05	-0.08	0.00	-0.30	0.79	0.00
2O	-0.01	0.02	0.00	0.14	-0.35	0.00	0.14	-0.39	0.00
3O	0.10	-0.02	0.00	-0.13	0.45	0.00	0.08	-0.19	0.00
4H	-0.77	0.45	0.00	-0.31	-0.11	0.00	0.04	-0.25	0.00
5H	-0.05	-0.43	0.00	-0.35	-0.63	0.00	0.00	0.03	0.00

Table S2. *Normal coordinates for reactive complex in its VTS geometry (reaction with D_2)*

	X	Y	Z	X	Y	Z	X	Y	Z
	-181 cm-1			232 cm-1			578 cm-1		
1C	-0.03	0.00	0.00	-0.06	0.02	0.00	0.00	0.00	0.87
2O	0.04	0.01	0.00	0.10	0.09	0.00	0.00	0.00	-0.35
3O	-0.09	-0.04	0.00	-0.13	-0.01	0.00	0.00	0.00	-0.30
4H	0.53	0.68	0.00	-0.21	-0.35	0.00	0.00	0.00	0.08
5H	0.00	-0.49	0.00	0.79	-0.41	0.00	0.00	0.00	-0.17
	585 cm-1			650 cm-1			776 cm-1		
1C	0.54	0.22	0.00	0.00	0.00	0.04	0.03	0.06	0.00
2O	-0.22	-0.07	0.00	0.00	0.00	-0.01	-0.02	0.07	0.00
3O	-0.13	-0.08	0.00	0.00	0.00	0.03	0.03	0.04	0.00
4H	-0.59	0.28	0.00	0.00	0.00	-0.88	0.26	-0.57	0.00
5H	0.11	-0.39	0.00	0.00	0.00	0.47	-0.49	-0.60	0.00
	882 cm-1			1270 cm-1			2383 cm-1		
1C	-0.12	-0.05	0.00	0.10	-0.13	0.00	-0.31	0.81	0.00
2O	0.02	0.01	0.00	0.22	-0.56	0.00	0.15	-0.40	0.00
3O	0.18	0.05	0.00	-0.27	0.69	0.00	0.08	-0.19	0.00
4H	-0.72	0.36	0.00	-0.07	-0.07	0.00	0.01	-0.12	0.00
5H	-0.14	-0.52	0.00	-0.14	-0.20	0.00	0.01	0.01	0.00

Table S3. *Cartesian coordinates of reagents, products and VTS*

		X	Y	Z
VTS	1C	0.000	0.000	0.000
	2O	0.000	0.000	1.144
	3O	0.000	0.095	-1.218
	4H	0.000	-0.913	-1.900
	5H	0.000	-1.772	-2.623
CO_2^+	O	0.000	0.000	0.000
	C	0.000	0.000	1.182
	O	0.000	0.000	2.364
H_2	H	0.000	0.000	-0.750
	H	0.000	0.000	0.000
HCO_2^+	O	0.000	0.000	0.000
	C	0.000	0.000	1.138
	O	-0.134	0.000	2.360
	H	0.660	0.000	2.949

2. Normal Mode Analysis of the Vibrational Frequencies of the VTS Geometry

Table S4. Normal mode analysis of the vibrational frequencies of the complex at its VTS geometry. In light-gray is highlighted the reaction coordinate, in dark-gray the normal modes coming from the CO_2, in white the intermolecular frequencies. Frequencies are in cm^{-1}.

**H_2**	**D_2**	**Normal mode**
-245	-181	*Reactive coordinate*
311	232	*O-H-H in plane bending*
578	578	*O-C-O bending*
608	585	*O-C-O bending*
909	650	*O-H-H out of plane bending*
1068	776	*O-H-H in plane bending*
1170	882	*H-O-C bending*
1283	1270	*O-C-O sym stretching*
2386	2383	*O-C-O asym stretching*

3. Average Internal Energy Distribution upon Photoionization

Following the same procedure reported in parag. 1.2 and 2.1 of the SI of the work Satta et al. ChemPhysChem 21 (2020) 1146-1156 we use the equations (1) and (2) and the measured threshold photoelectron spectra (TPES) cross-sections σ_j [1,2,3] to obtain the average internal (E^{el}_{INTER}) and kinetic (E^{el}_{Kin}) energy distribution upon photoionization of CO_2 shown in Figure S1 :

$$E^{el}_{INTER}(h\nu) = \frac{\sum_j \sigma_j(h\nu)(h\nu_j - IE)}{\sum_j^n \sigma_j(h\nu)} \quad (1)$$

$$E^{el}_{Kin}(h\nu) = \frac{\sum_j \sigma_j(h\nu)(h\nu - h\nu_j)}{\sum_j \sigma_j(h\nu)} \quad (2)$$

and equation (3) to obtain the Rate Coefficients as a function of photon energy.

$$k(h\nu) = \frac{ln[R(h\nu)+1]}{n_{H2}\tau_R} \quad (3)$$

Where R is the product-reagent ratio at the characteristic detection time τ_R and n_{H2} is the number density of hydrogen inside the cell. The rate coefficient at T=298 K was taken as $5.80 \times 10^{-10} \pm 10\%$ molecule^{-1}s^{-1}cm^{3},[4] for H_2 and $4.10 \times 10^{-10} \pm 10\%$ molecule^{-1}s^{-1}cm^{3} for D_2, and at P=$6.5 \times 10^{-5} \pm 30\%$ mbar, the characteristic detection time τ_R is 1.44±0.60 ms, for H_2 and 1.90±0.80 ms for D_2. From Figure S1 it is clear that almost all the photoionization energy goes to the kinetic energy of the electron, and at hν=15 eV only a small energy content of 0.13 eV is stored in the internal vibrational degrees of freedom of the CO_2^+ ion.

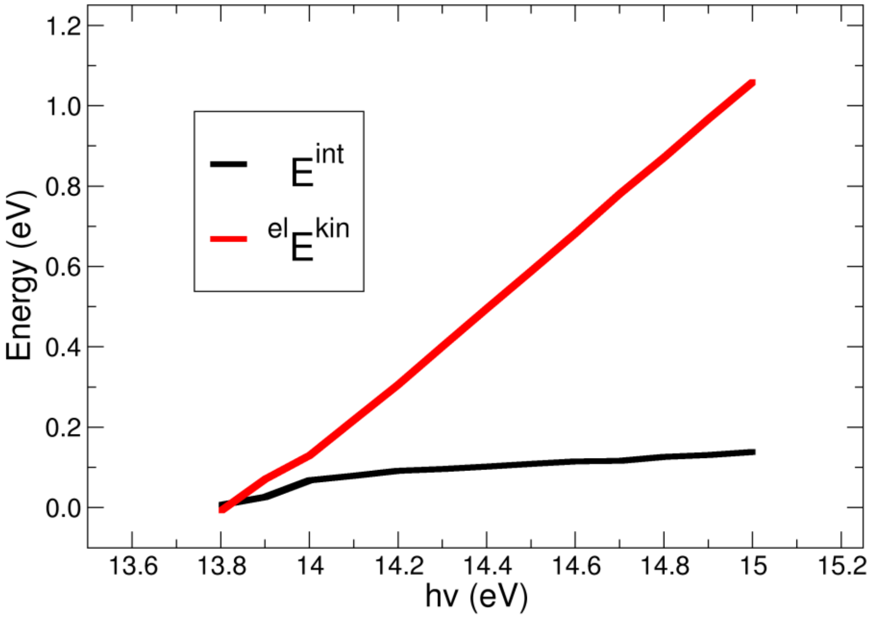

Figure S1. Average internal energy of the CO_2^+ ion (black line) and average kinetic energy of the photoelectron (red line) as a function of the photoionization energy hν.

4. Rate Coefficients for the Reaction of CO_2^+ with H_2 and D_2 in a range of X_aX_b pair

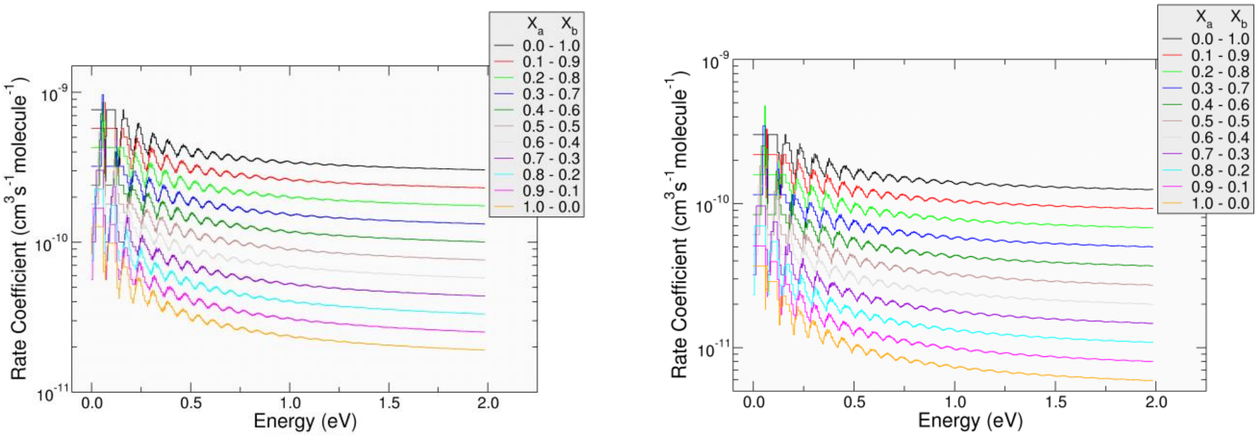

Figure S2. Rate coefficients for a range of X_aX_b values for the reaction of CO_2^+ with H_2 (on the left) and D_2 (on the right). The data are referred to energy flow fraction X(300K)=0.0612 and X(300K)=0.0653 for H_2 and D_2, respectively.

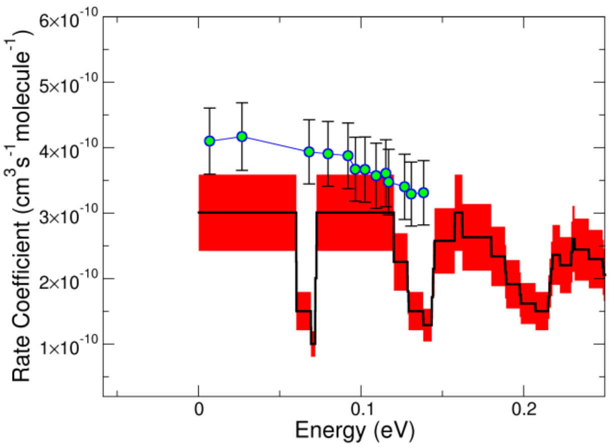

Figure S3. Theoretical and experimental rate coefficients as a function of the internal energy of CO_2^+ in the reaction with D_2. The cyan circles represent the experimental data, while the black line is the theoretical rate coefficient calculated at X_a=0.0, X_b=1.0. The energy flow is such that X(300K)=0.0653, The red area shows the uncertainty due to the 20% error on the experimental rate coefficient from Gerlich.[5]

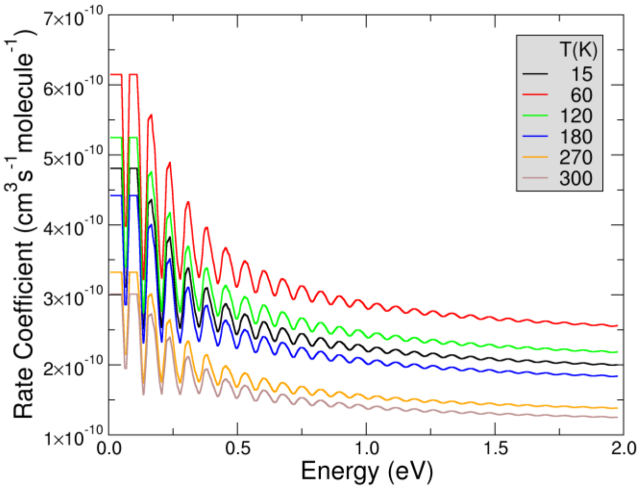

Figure S4: Rate coefficients for the deuterium transfer reaction between CO_2^+ and D_2 as a function of the internal energy acquired during the photoionization of the CO_2^+. T is the temperature of the roto-translations of the CO_2^+ and of the vibro-roto-translation of the D_2 reagents. The energy flow is such that X(300K)=0.0653, and X_b=1.0.

5. Rate Coefficients versus Temperature of the Reactants

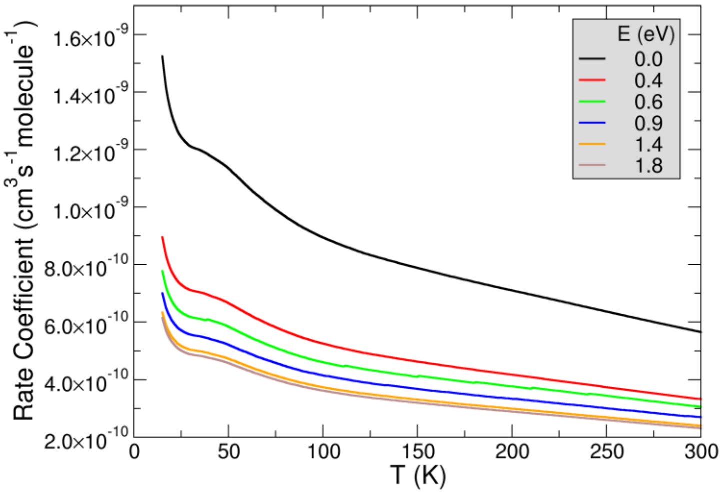

Figure S5: Rate coefficient for the reaction of CO_2^+ with H_2 as function of temperature. Data are referred to different energy content of the CO_2^+ ion. The temperature is that of the roto-translations of the CO_2^+ and of the vibro-roto-translation of the H_2 reagents.

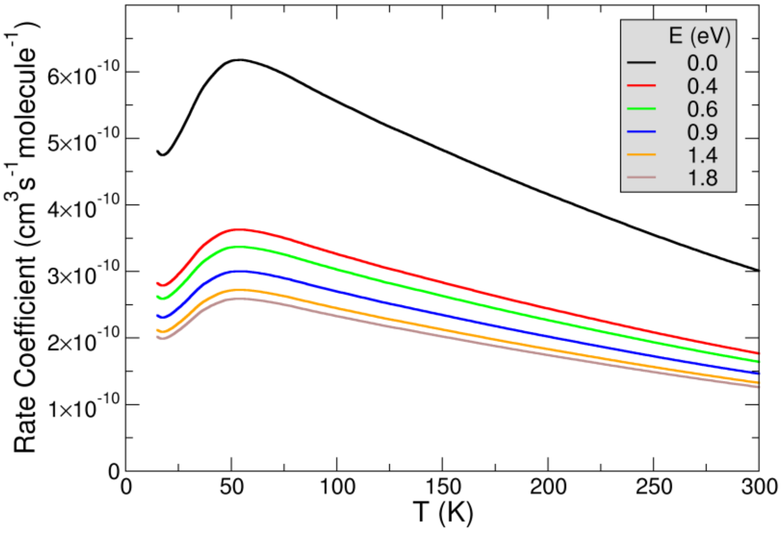

Figure S6: Rate coefficient for the reaction of CO_2^+ with D_2 as function of temperature. Data are referred to different energy content of the CO_2^+ ion. The temperature is that of the roto-translations of the CO_2^+ and of the vibro-roto-translation of the D_2 reagents.

[1] J. Liu; W. Chen, C.-W. Hsu; M. Hochlaf; M. Evans, S. Stimson; C. Y. Ng “High Resolution Pulsed Field Ionization−Photoelectron Study of CO_2 ($X^2\Pi_g$) in The Energy Range of 13.6−14.7 eV. *J. Chem. Phys.* **2000**, *112*, 10767−10777.

[2] A .W. Potts and G. H. Fattahallah “High-resolution ultraviolet photoelectron spectroscopy of CO_2, COS and CS_2” *J. Phys. B: Atom. Mol. Phys.* **1980**, *13*, 2545.

[3] T. Baer and P. M. Guyon “Autoionization and isotope effect in the threshold photoelectron spectrum of $^{12}CO_2$ and $^{13}CO_2$” *J. Chem. Phys.* **1986**, *85*, 4765.

[4] V. G. Anicich An Index of the Literature for Bimolecular Gas Phase Cation-Molecule Reaction Kinetics **2003** JPL Publication 03-19.

[5] G. Borodi, A. Luca, D. Gerlich “Reactions of CO_2^+ with H, H_2 and deuterated analogues” *Int. J. Mass Spectrom.* **2009**, *280*, 218–225.
